# Antibacterial Peptide Nucleic Acids—Facts and Perspectives

**DOI:** 10.3390/molecules25030559

**Published:** 2020-01-28

**Authors:** Monika Wojciechowska, Marcin Równicki, Adam Mieczkowski, Joanna Miszkiewicz, Joanna Trylska

**Affiliations:** 1 Centre of New Technologies, University of Warsaw, Banacha 2c, 02-097 Warsaw, Poland; 2 College of Inter-Faculty Individual Studies in Mathematics and Natural Sciences, University of Warsaw, Banacha 2c, 02-097 Warsaw, Poland; 3 Institute of Biochemistry and Biophysics, Polish Academy of Sciences, Pawińskiego 5a, 02-106 Warsaw, Poland

**Keywords:** oligonucleotides, peptide nucleic acid (PNA), antibacterials, RNA, PNA transporters, conjugates, bacterial resistance

## Abstract

Antibiotic resistance is an escalating, worldwide problem. Due to excessive use of antibiotics, multidrug-resistant bacteria have become a serious threat and a major global healthcare problem of the 21st century. This fact creates an urgent need for new and effective antimicrobials. The common strategies for antibiotic discovery are based on either modifying existing antibiotics or screening compound libraries, but these strategies have not been successful in recent decades. An alternative approach could be to use gene-specific oligonucleotides, such as peptide nucleic acid (PNA) oligomers, that can specifically target any single pathogen. This approach broadens the range of potential targets to any gene with a known sequence in any bacterium, and could significantly reduce the time required to discover new antimicrobials or their redesign, if resistance arises. We review the potential of PNA as an antibacterial molecule. First, we describe the physicochemical properties of PNA and modifications of the PNA backbone and nucleobases. Second, we review the carriers used to transport PNA to bacterial cells. Furthermore, we discuss the PNA targets in antibacterial studies focusing on antisense PNA targeting bacterial mRNA and rRNA.

## 1. Introduction

Excessive use of antibiotics has led to an alarming situation when many bacterial strains developed resistance to these antibiotics. According to the World Health Organization, resistance to existing antibiotics, and slow rate of developing their new classes are currently among the greatest threats for human health [1,2]. Bacteria are particularly dangerous because they have already acquired resistance to several antibiotics at once, which has led to multi-drug resistance strains (MDR). The MDR among clinical isolates have made the current antibiotics inefficient, which, in turn, has increased the spread of resistant bacteria [3]. In the light of these facts, development of new potent antimicrobial agents is extremely necessary [4]. Long development times and high costs limit the discovery of new antimicrobial agents, so the most effective antibiotics are based on modifications of the previously discovered ones [5]. Thus, we urgently need new antibiotic types with a new mechanism of action.

Antisense oligonucleotides, used to inhibit the synthesis of proteins essential for bacteria to sustain life, may be helpful in the fight against bacterial infections. One such oligonucleotide is the peptide nucleic acid (PNA) molecule that combines the properties of both peptides and nucleic acids. PNA were designed as synthetic analogues of DNA [6], which contain a neutral backbone, are resistant to enzymes degrading proteins [7] and nucleic acids [8] and form stable complexes with DNA and RNA. PNA oligomers are synthesized on solid support with a simple method similar to that used to synthesize peptides. This method, known as solid-phase peptide synthesis (SPPS), has been well described in the literature [9].

Inside bacteria, antisense PNA oligomers inhibit the translation process by binding to mRNA or the ribosome. The antisense effect of PNA is based on the formation of hydrogen bonds between the complementary PNA sequence and selected nucleic acid target. An important advantage of PNA is its selectivity and high-affinity binding. Thanks to that, it is possible to design PNA-based antimicrobials specific for particular genes in selected bacteria. In principle, PNA show huge potential to control the spread of resistant microorganisms. Unfortunately, the use of PNA in antibacterial applications encountered several crucial obstacles. The hydrophobicity of the PNA backbone causes problems with PNA solubility in aqueous solutions, which leads to PNA adopting compact structures susceptible to aggregation [10]. One of the consequences of PNA poor water solubility is difficulty in the delivery of PNA oligonucleotides to bacterial cells [11]. Several strategies of improving the PNA solubility in water and increasing PNA uptake by bacteria have been proposed [12,13]. In this review, we have summarized and presented these strategies. In the last decade, a few reviews on PNA antibacterial applications have been published, e.g., [14,15,16,17,18]. We have updated this information, specifically focusing on PNA modifications, structural data for PNA-involving complexes, antibacterial targets, and transport into bacterial cells.

## 2. PNA Complexes with Natural Nucleic Acids

To point-out the antibacterial potential of PNA and challenges facing any future therapeutic applications of these molecules, it is necessary to understand the structural and physicochemical properties of PNA. In this section, we present the most relevant PNA properties and structural fundaments of PNA complexes with nucleic acids.

Besides the higher enzymatic stability, PNA has another important advantage: it hybridizes with complementary sequences of natural nucleic acids creating either duplexes or triplexes. So far, nearly 20 structures containing PNA oligomers have been solved by X-ray crystallography or nuclear magnetic resonance (NMR) including single-stranded PNA, PNA-PNA, PNA-DNA and PNA-RNA duplexes, and a triplex of double-stranded PNA with DNA (summarized in Table 1).

The simplest duplexes observing the Watson–Crick base-pairing scheme are formed by single-stranded PNA with complementary strands of DNA [31,32,33], RNA [28,30], or PNA [20,21,22,23,24,25,26,27,28] (Table 1). In these structures the single-strand of PNA (6–11 monomers), typically of a mixed sequence, binds to DNA or RNA strands in an antiparallel way (C_term_-PNA to 5′-DNA/RNA, N_term_-PNA to 3′-DNA/RNA). In most crystallized duplexes, the PNA terminus is extended with a lysine.

However, in general, classical PNA duplexes can be formed both in a parallel and antiparallel manner. Also, such PNA duplexes can form right- and left-handed P-type helices, characterized by a deeper and wider major groove, smaller angle, and larger displacement as compared to typical DNA and RNA helices. The P-type helix is 28 Å wide and, for comparison, classical helices composed of natural oligonucleotides are 23 Å (in the case of an A-helix) and 20 Å (B-helix) wide. The P-type helix has 18 base pairs per turn (as compared to A-helix – 11 and B-helix – 10). The PNA-DNA or PNA-RNA hybrids tend to be organized as B- or A-like helices, respectively [23,34].

**Figure 1:**
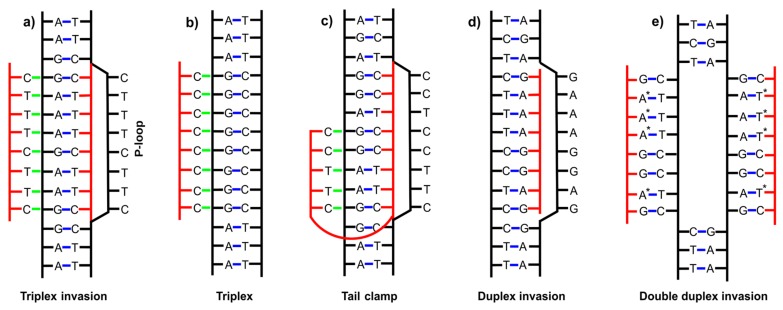
Scheme showing the examples of complexes of PNA with double-stranded DNA: (**a**) triplex invasion, (**b**) triplex, (**c**) tail clamp, (**d**) duplex invasion, (**e**) double duplex invasion. Red lines —PNA backbone; black lines—DNA; blue dashed lines—Watson–Crick hydrogen bonds; green dashed lines—Hoogsteen-type hydrogen bonds; *—modified nucleotide bases [35].

In addition to forming duplexes, single-stranded PNA can also bind to double-stranded DNA or RNA. Homopyrimidine PNA has the ability to bind a homopurine strand of a DNA duplex, opening the DNA helix and displacing the non-complementary DNA strand that forms the so-called P-loop [35]. As a result, a stable and thermodynamically favorable triplex-invasion complex is acquired (Figure 1a) [36]. If homopyrimidine PNA is rich in cytosines, it binds a DNA duplex without strand-displacement forming a classical triplex (Figure 1b). Notably, classical triplex can be also formed by binding a single strand of DNA to a PNA duplex. One such triplex has been crystallized by Betts et al. [34]; a homopurine DNA strand created a triplex with a homopyrimidine PNA hairpin (Table 1). The ability of PNA to create triplexes enables the formation of the so-called bis-PNA (a double-stranded PNA formed via e.g., an ethylene glycol type linker) [37,38] with two strands of DNA creating a tail clamp structure (Figure 1c). If PNA is a homopurine strand, a duplex invasion complex (Figure 1d) with a DNA duplex is created [39]. Moreover, under special circumstances, pseudo-complementary PNA strands with modified nucleobases—e.g., diaminopurine, thiothymine, and thiouracil—do not recognize each other due to steric hindrance and bind simultaneously to a double-stranded DNA forming a double duplex invasion complex (Figure 1e) [40,41]. In conclusion, five different modes of binding of PNA to double-stranded DNA have been found showing a wide and diverse capability of PNA to form complexes (Figure 1) [35].

Apart from NMR and crystallography, the complexes with PNA have been investigated also by other experimental methods, e.g., isothermal titration calorimetry [42,43], differential scanning calorimetry (DSC) [44], circular dichroism (CD) spectroscopy [45,46], UV-monitored thermal melting [44,45,47], fluorescence spectroscopy [46,47], gel electrophoresis [48,49], and nano-electrospray ionization mass spectrometry [46]. Computational methods, such as molecular dynamics simulations of single-stranded PNA [50,51,52] and of PNA-involving complexes [45,53,54,55] have been also performed giving insight into PNA (thermo)dynamics at atomistic level of detail.

PNA are achiral molecules, but chiral centers can be introduced by adding amino acids into the PNA oligomer or at its terminus (typically a lysine is added). As a result, CD can be observed confirming the helicity of PNA-PNA, PNA-DNA, and PNA-RNA duplexes [56].

**Figure 2:**
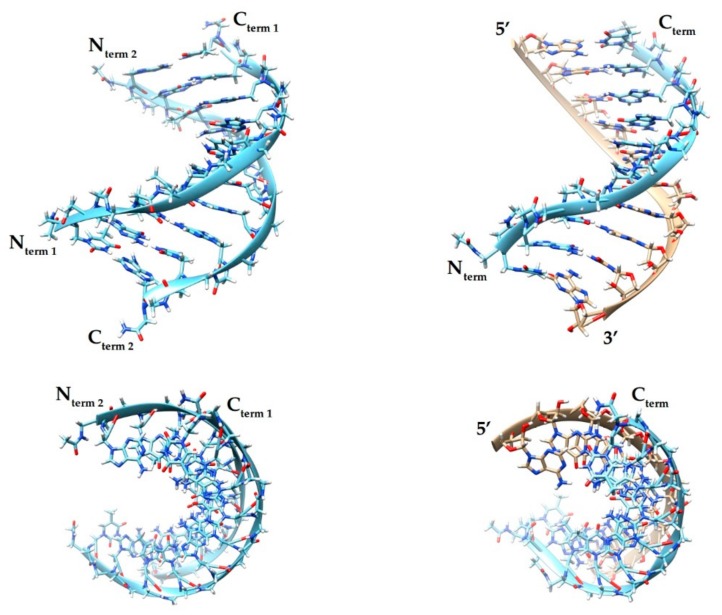
Side and top views of a PNA-PNA (left) and PNA-RNA (right) tertiary structures from molecular dynamics simulations [45]. The figure was made using Chimera 1.12 [57]. Light blue—PNA strands; beige—RNA; dark blue—nitrogen; red—oxygen; white—hydrogen.

Using UV spectroscopy complemented with molecular dynamics simulations, the melting temperature (T_m_) profiles of PNA-PNA and PNA-RNA 10-mer mixed-sequence duplexes were determined (Figure 2) [45]. The results showed that T_m_ of the PNA-PNA duplex is higher than that of PNA-RNA by about 1.5 degrees per base pair. Molecular dynamics simulations of melting at atomistic level of detail suggested that a PNA duplex ‘melts’ cooperatively over its entire length, while PNA-RNA preferentially melts starting from the termini.

The types of complexes presented in Figure 1 depend not only on the sequence composition of the nucleic acid strands but also on many other factors such as the sequence length, the number of mismatches, the modifications introduced to PNA, environmental conditions such as buffer composition and ion concentration. Considering all these factors upon designing a PNA sequence for a particular application is not straightforward because our knowledge is limited. Thus, despite the large amount of work already put into the studies of PNA complexes with natural nucleic acids, many questions still remain unanswered and predictions of PNA binding affinities, especially to more complex RNA tertiary structures, are not evident.

## 3. Chemical Modifications of PNA

**Figure 3:**
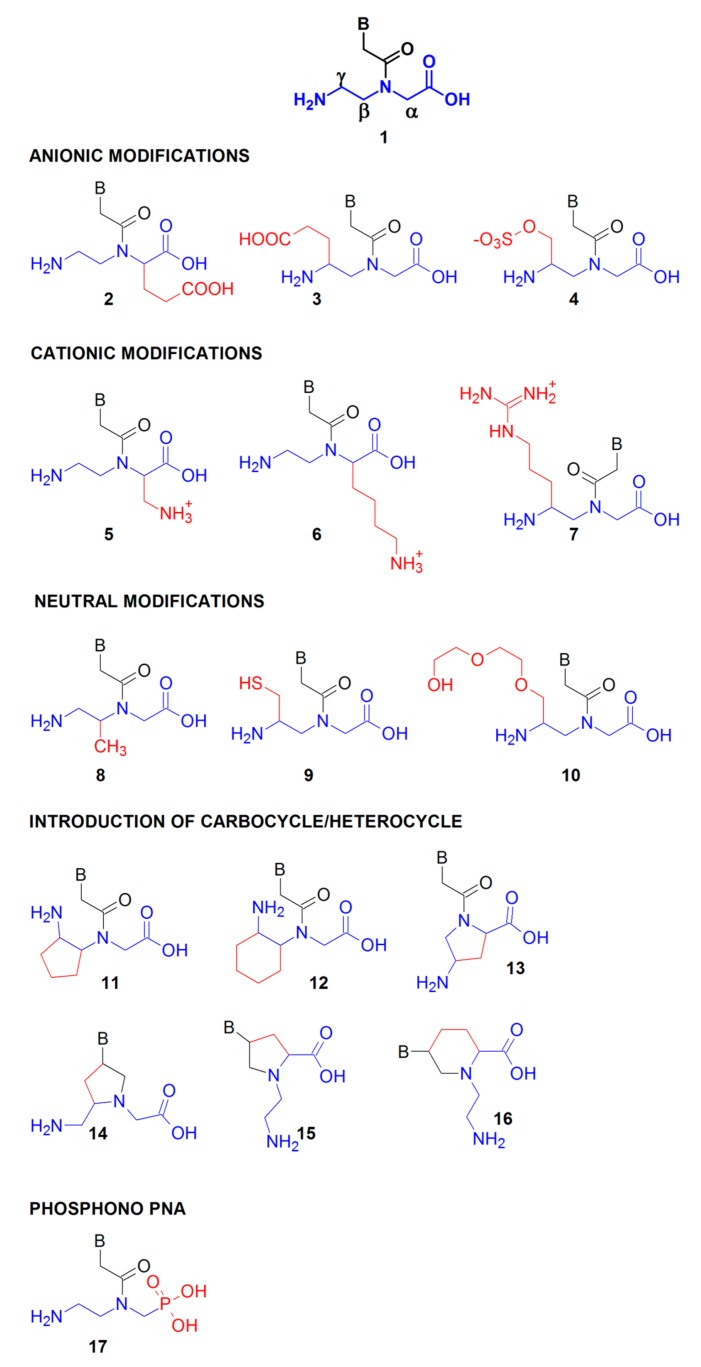
Selected modifications of the PNA backbone; the *N*-(2-aminoethyl)glycine backbone with the α-, β-, or γ-position is shown in blue and the introduced modifications are shown in red. B stands for adenine, cytosine, guanine, or thymine.

To improve PNA solubility or affinity toward natural nucleic acids, PNA peptide-like backbone has been further modified. Many structural modifications were introduced to change the properties of the PNA scaffold (**1**, see Figure 3 for numbering of scaffolds) including variations in length, type and functionalization of the peptide-like backbone, the type and length of the linker connecting the heterocyclic base to the backbone, as well as the type and functionalization of heterocyclic moieties. Modifications of the *N*-(2-aminoethyl)glycine backbone in the α-, β- or γ-position (Figure 3) result in a new stereogenic center, thus chiral PNA are formed [58]. Modifications introduced in the γ-position of the PNA backbone improved hybridization properties as compared to those introduced in the α-position (Figure 3) [13].

The substituents incorporated into the PNA monomer backbone can be anionic, through introduction of the carboxylic **2**, **3** [59], sulphate **4** group [60] or cationic **5**, **6**, **7**. The cationic α-aminomethylene **5** [61], α-lysine **6** or guanidine **7** [62] in the PNA backbone enhanced cellular uptake and increased the stability of nucleic acid duplexes involving PNA. Furthermore, neutral moieties were also introduced including α-methyl **8** [63], γ-methylthiol **9** [64], or γ-diethyleneglycol—“miniPEG” **10** [65] to modulate other PNA properties such as aggregation propensity, water solubility, sequence selectivity, and nucleic acid affinity. Preorganization of the PNA structure was achieved by introducing cyclic rigid moieties possessing carbocyclic cyclopentyl **11** [66], cyclohexyl **12** [67], or heterocyclic pyrrolidine scaffolds **13** [68,69]. Additionally, by introducing a linker between the heterocyclic group and peptide backbone, rigid, heterocyclic scaffolds based on pyrrolidine ring **14** [70,71] and **15** [72] as well as piperidine **16** [73] ring were developed. Finally, phosphono PNA, bearing phosphonoamidate bonds were synthesized from the appropriate phosphonate unit **17** [74].

**Figure 4:**
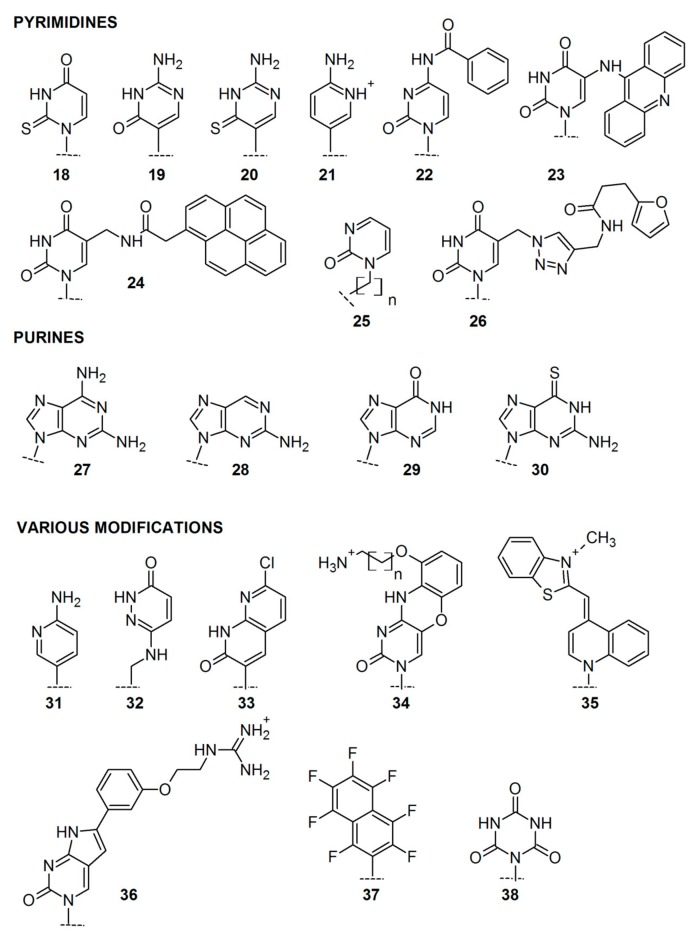
Selected modifications of nucleobases in PNA monomers.

To further modulate the properties of PNA oligomers [75], different nucleobase modifications were also developed, including modifications of functional groups in purine/pyrimidine bases and modifications of the heterocyclic core itself (Figure 4). Modified bases in the PNA monomers increased PNA affinity and selectivity, enhanced duplex stability and recognition, as well as triplex formation. In many cases, they also enabled monitoring PNA fluorescence.

The most common non-coding pyrimidine bases introduced in PNA include 2-thiouracil **18**, used for the development of pseudo-complementary PNA [76,77], pseudoisocytosine **19** [78], thio-pseudoisocytosine **20** [49], and 2-aminopyrimidine **21** [79] for stable triplex formation with RNA duplexes. *N*^4^-benzoylcytosine **22** was introduced by the Nielsen group [80,81] as a candidate for a pseudo-complementary G-C base pair, and 5-(acridin-9-ylamino)uracil **23** was applied as fluorescent, hydrolytically labile nucleobase modification [82]. Manicardi et al. studied the pyrene-labeled, fluorescent PNA monomer **24** [83] and used it to investigate stacking interactions and selective excimer emission in PNA_2_/DNA triplexes. 2-pyrimidinone as a nucleobase **25** was introduced to short PNA, which bound strongly to a homopurine tract of complementary RNA [84], while furan-modified uracil derivative **26** was designed as a mildly inducible, irreversible inter-strand crosslinking system targeting single and double-stranded DNA [85]

Modifications of purine bases led to the development of 2,6-diaminopurine **27** applied to the design of pseudo-complementary PNA [86,87], 2-aminopurine **28** used as a fluorescent probe for examining PNA–DNA interaction dynamics [87,88], hypoxanthine **29**, which could form Watson–Crick base pairs with adenine, cytosine, thymine, and uracil increasing the specificity of PNA [89,90], and 6-thioguanine **30**, which caused helix distortion at the 6sG:C base pair, but the base stacking throughout the duplex was still retained [91].

Finally, diverse heterocyclic bases were introduced in the place of either purine or pyrimidine bases. 2-Aminopyridine **31** was applied for the triplex-forming PNA [92], 3-oxo-2,3-dihydropyridazine monomer **32** was introduced to the PNA oligomer to increase affinity and selectivity of modified PNA to a microRNA [93]. Bicyclic 7-chloro-1,8-naphthyridin-2(1*H*)-one **33** turned out to be an effective thymine substitute in the PNA oligomers and increased PNA affinity in both duplex and triplex systems [94]. Introduction of tricyclic phenoxazine analog, 9-(2-aminoethoxy)phenoxazine (G-clamp) **34**, enhanced the stability of PNA complexes with target nucleic acids [95,96]. Incorporation of the fluorescent dye, Thiazole Orange **35**, enabled detection of homogeneous single nucleotide mutations [97]. One of the pyrrolocytosine bases **36** exhibited increased selectivity, binding affinity, and high fluorescence quantum yield in response to PNA hybridization [98]. Moreover, fluoroaromatic universal bases including **37** [99] and cyanuric acid derivatives as nucleobases **38** were applied to decrease base pairing discrimination by PNA probes, which could be desirable in some diagnostic applications [100].

Thanks to these advances in PNA chemistry, a number of modified PNA with properties better suited for biological applications have been presented. The aim of these changes was mainly to improve PNA affinity to natural nucleic acids, solubility, and membrane permeability. So far, no studies have been conducted with modified PNA oligomers as antibacterials. Although many new PNA analogs have been synthesized, still classical PNA monomers are most commonly used providing a reasonable balance between the requirement of high affinity for natural nucleic acids and specificity of the sequence recognition. Considering the problem of PNA delivery into bacteria, the most promising seem to be modifications that introduce positively charged groups into the PNA skeleton (compounds **5**, **6**, **7**). Introduction of cationic groups into PNA should also improve its solubility and affinity to negatively charged nucleic acids.

Up to now, the γ-modified PNA was used as a diagnostic tool for identification of bacterial and fungal pathogens in blood [101]. This is one of the possible ways of using modified PNA in pathogen diagnostics. Furthermore, compared to conventional monomers, γ-PNA have several advantages: increased stability of duplexes with nucleic acids, better solubility, and less self-aggregation. Therefore, γ–modified PNA (e.g., compound number **3**, **4**, **7**, **9**, **10**) could be potentially useful also in antibacterial applications.

## 4. Delivery of PNA to Bacteria

In order to block the expression of a specific gene, PNA must first enter the bacterial cell. Unfortunately, PNA does not have the ability to spontaneously permeate bacterial membranes. Due to different transport mechanisms, effective delivery of PNA to bacteria is much more difficult than its delivery to mammalian cells. The main limitations hindering the development of antimicrobial PNA are poor PNA solubility in aqueous solutions, the lack of bacterial membrane permeability by PNA, and the associated difficulty of finding effective transporters of PNA to bacterial cells.

The cell wall of bacteria is an effective barrier for foreign particles, including PNA. Good et al. demonstrated that, in gram-negative bacteria, the main barrier for PNA is the lipopolysaccharide (LPS)—a component of the outer cell membrane [102]. They proved that *Escherichia coli* (*E. coli*) strains with defective LPS were more sensitive to PNA than strains without this modification. Overall, the antibacterial potential of PNA increased if *E. coli* was cultured in the presence of factors increasing cell wall permeability. However, PNA activity did not improve after introducing mutations in the genes encoding efflux pumps responsible for antibiotic resistance, suggesting that PNA is not a substrate for these pumps [102].

**Figure 5:**
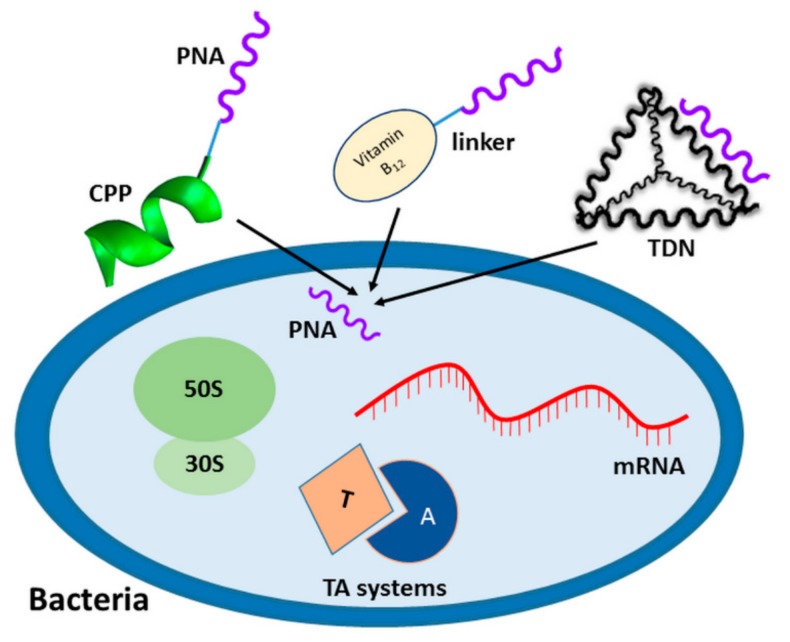
Schematic representation of PNA delivery strategies to bacterial cells: covalent conjugation of PNA with CPP or vitamin B_12_, and complementary base pairing between PNA and DNA in tetrahedral DNA nanostructure (TDN). PNA targets tested in bacteria: mRNA, ribosome, and toxin–antitoxin (TA) systems are also shown.

As mentioned above, poor water solubility and difficulty in delivering PNA to the cell interior are the major constraints in any PNA applications. Different strategies have been proposed to improve PNA bioavailability. One of them includes chemical modifications of the PNA backbone to increase PNA hydrophilicity (see the section on PNA modifications, e.g., the compound number **6** or **7**, Figure 3). Another strategy is based on the conjugation of a PNA oligomer to positively charged amino acids at the PNA terminus [103,104]. An alternative is combining PNA with molecules capable of penetrating bacterial cells, which act as PNA transporters (Figure 5). In this section, we summarize available PNA delivery strategies used to achieve antimicrobial effects.

Until now, the most effective way of transporting PNA to bacteria was by cell penetrating peptides (CPP), (Figure 5) [105]. CPP are short (usually consisting of less than **30** amino acids) cationic or amphipathic peptides that can transport molecules many times their weight. There are two ways to combine antisense oligonucleotides with CPP. One is the conjugation of a CPP with an oligonucleotide through a covalent bond, and the other one is the formation of a non-covalent complex [106]. Most CPP and PNA conjugates proposed so far are covalently linked.

The mechanism of cell penetration by CPP may be different for different bacteria. The most commonly used CPP that transports PNA into bacterial cells is the synthetic peptide (KFF)_3_K, which was first synthesized by Vaara and Porro in 1996 [107] based on the skeleton of the antibiotic polymyxin B. (KFF)_3_K efficiently transports PNA in vitro, both to gram-negative and gram-positive cells [108]. Despite its efficiency in vitro, the activity of (KFF)_3_K-PNA conjugates drastically decreases in the presence of blood serum [109]. Moreover, this peptide causes hemolysis at concentrations above 32 µM [107]; for comparison, polymyxin B is not hemolytic up to 1100 µM. Therefore, (KFF)_3_K is not an ideal candidate for a PNA transporter and its future medical use is doubtful.

Several other CPP have been tested as PNA carriers in vitro, including (RXR)_4_XB (X—6-aminohexanoic; B—β-alanine) [110], the TAT peptide produced by human immunodeficiency virus [111], and many others [112,113]. Abushahba et al. [112] tested the antibacterial effect of PNA attached to five different CPP. In this work, PNA inhibited the *rpoA* gene, which is the key gene for the survival of *Listeria monocytogenes*. The authors confirmed that (RXR)_4_XB, TAT and (RFR)_4_XB, are the most effective in introducing PNA into *L. monocytogenes*. The same peptides were tested by the Patenge group [105] and conjugated to PNA complementary to the fragment of the *gyrA* gene in order to inhibit the growth of *Streptococus pyogenes*. Out of 18 different peptides, TAT, oligolysine (K8), and (RXR)_4_XB, effectively inhibited the growth of the tested strains.

The first protein identified as involved in the transport of peptide-PNA conjugates is an inner membrane protein SbmA [114]. Ghosal et al. have shown that first, the peptide-PNA conjugate passes through the outer membrane, then the peptide carrier is degraded by proteases, and next, SbmA is involved in the transport of the free PNA through the inner membrane. However, in another work, it was shown that the SbmA protein is not always required for antibacterial activity of the peptide-PNA conjugate [115]. Hansen et al. tested 16 conjugates of PNA with antimicrobial peptides. They identified three SbmA-independent, antimicrobially active PNA conjugates with peptides: Pep-1-K, KLW-9,13-α and drosocin-RXR. In addition, in [116] it was shown that the involvement of SbmA in the peptide-PNA transport also depends on the length of the PNA oligomer.

The effectiveness of PNA delivery into bacteria using CPP can be modulated by the linker between the PNA and peptide (using either a degradable or non-degradable one) [117,118]. The most commonly used linker in the CPP-PNA conjugates is a flexible ethylene glycol linker [105,116]. Good et al. [11] compared two antibacterial CPP-PNA conjugates with the same sequence but different linkers (degradable, maleimide; and non-degradable, ethylene glycol). They showed that the conjugate with the degradable linker is 10 times less active against *E. coli* than the conjugate with the ethylene glycol linker. Many other linkers were tested, e.g., a stable triazole ring [119] or degradable disulfide bond [117,120]. We found that the conjugates with the ethylene glycol linker showed improved antimicrobial activity as compared to the same conjugates but connected through the triazole ring (unpublished observation). A comprehensive comparison of different linkers in peptide conjugates with different oligonucleotides can be found in the review [121].

Note, that CPP as PNA carriers are not universal because the transport of CPP may be strain dependent. Additional obstacle in the use of CPP is that they may be cytotoxic to eukaryotic cells and cause hemolysis of erythrocytes [107,122]. Therefore, there is still a need to develop effective and noninvasive methods of introducing short, modified oligonucleotides (such as PNA oligomers) into bacterial cells.

Beyond CPP, few non-peptidic molecules have been investigated to actively transport PNA to bacteria. One such carrier of PNA is vitamin B_12_ (Figure 5). All aerobic bacteria require vitamin B_12_ for growth, but only a few produce it de novo [123] therefore, most microorganisms are forced to take up vitamin B_12_ from the environment. In recent years, PNA was combined with vitamin B_12_ using different linkers. Vitamin B_12_ was also found to improve PNA solubility and make the PNA in the conjugate adopt a more extended conformation in comparison with free single-stranded PNA [124]. Furthermore, we have shown that vitamin B_12_ acts as a carrier of PNA to *E. coli* and *Salmonella enterica subsp. enterica serovar* Typhimurium [119,125]. These studies indicate that vitamin B_12_ could be a good candidate for a PNA transporter into bacteria. However, the concentrations of vitamin B_12_ required for bacterial growth are smaller than the concentrations of PNA that are necessary to exert an antibacterial effect.

An interesting and innovative approach was proposed in the work Readman et al. [126]. The self-assembling three-dimensional structure of a DNA tetrahedron was used as a carrier for PNA oligomers into *E. coli* (Figure 5). The authors developed a DNA tetrahedron vector based on a single-stranded DNA incorporating a PNA into its structural design. The PNA-tetrahedral DNA nanostructure (TDN) inhibited bacterial growth at lower concentrations than the previously reported (KFF)_3_K-PNA conjugate [126]. The transport mechanism of such complexes is not clear and further studies of this vector are needed. Nevertheless, TDNs are promising candidates for PNA vectors because they are non-toxic to cells as compared to CPP. In another work [127], TDNs efficiently transported antisense PNA (targeting the *ftsZ* gene) into methicillin-resistant *Staphylococcus aureus*.

Despite these few other strategies to deliver PNA to bacteria, the covalent conjugation of PNA and CPP is still the most popular one, mainly due to well-developed and relatively easy synthesis protocols. These protocols allow quick changes of the peptide sequence, PNA attachment site, and the linker type. However, this way of delivering PNA to bacteria is not ideal. Peptide uptake depends on bacterial strain and, at high concentrations, CPP exhibit toxicity to both bacterial and eukaryotic cells. Importantly, the new carriers such as vitamin B_12_ and TDN have proven that we do not have to limit ourselves to cationic peptides and completely different (non-peptidic) types of transporters could be considered and tested.

## 5. Applications of PNA as an Antibacterial Agent

In this section, we summarize the PNA sequences used as antibacterials. Since it is impossible to list all the PNA-targeted genes and strains, we overview the most promising reports presenting the lowest minimal inhibitory concentrations (MIC) necessary to inhibit bacterial growth. Table 2 summarizes the MIC values for different (KFF)_3_K-PNA conjugates aimed at various targets and bacterial strains. Note, that the summary in Table 2 is only indicative of PNA antibacterial activity because it is impossible to compare the effectiveness of different PNA sequences that target different stages of bacterial metabolism. Also, the (KFF)_3_K carrier may work differently in different strains. Similarly, it is impossible to compare the activity of PNA with classical antibiotics. Nevertheless, the MIC values in Table 2 are promising and motivate further research on antibacterial PNA.

### 5.1. Targeting the mRNA of Essential Genes with Antisense PNA

In the last two decades, many mRNA encoding essential genes in clinically pathogenic bacteria have been validated as possible targets for antisense PNA (Table 2). The most effective were PNA designed as complementary to mRNA around the start codon and its neighboring region. By binding to mRNA, PNA acts as a steric hindrance, contrary to some other oligonucleotides that induce the activity of RNase H.

The first reported antibacterial PNA targeted the mRNA transcript of *E. coli acpP* gene that encodes the acyl carrier protein, a protein crucial in fatty acid biosynthesis [11]. The *acpP* gene is conserved among gram-negative bacteria and has become a frequent PNA target in various human pathogens such as: *Brucella suis* [128], *Haemophilus influenza* [129], *Pseudomonas aeruginosa* [130]. In addition, a *fabI* gene, also involved in fatty acid biosynthesis, was targeted in *E. coli* and *S. aureus* [131]. The *E. coli* growth was also inhibited by PNA directed at mRNA involved in the folate biosynthesis (*folA* and *folP)* [131].

To improve the antimicrobial activity, PNA have also been used in combination with antibiotics. Dryselius et al. and Castillo et al. analysed synergistic interactions between PNA targeting fatty acid and folate synthesis pathways and a series of conventional antibiotics used against *E. coli* [131,132]. Antibiotics were selected based on their clinical relevance and targeted various biosynthetic pathways. These included aminoglycosides, penicillins, polymyxins, rifamycins, sulfonamides, and trimethoprim. The authors found several new synergistic combinations. Surprisingly, in both studies, higher synergy of action was reported for inhibitor combinations with functionally unrelated targets than for combinations with related targets.

Dryselius et al. examined the effects of the combinations of PNA and drugs against folate biosynthesis: sulfonamides that target dihydropteroate synthase (of the *folP* gene) in an early step of folate biosynthesis, and trimethoprim that inhibits dihydrofolate reductase (of the *folA* gene) in the later step of this pathway. They synergy was observed if the anti-*folA* PNA was combined with sulfamethoxazole, but no synergy was detected if anti-*folP* PNA was combined with trimethoprim [131]. Similarly, Castillo et al. demonstrated that PNA targeted at the essential *acpP* gene (involved in biosynthesis of fatty acids) exhibited synergistic interaction with trimethoprim, whose target is unrelated [132]. The molecular mechanisms of synergistic actions of these combinations are yet undiscovered and require further investigations. By contrast, Patenge et al. found antimicrobial synergy against *S. pyogenes* for the combination of anty-*gyrA* PNA with levofloxacin and novobiocin, agents that share the same target, namely the gyrase enzyme [111]. These synergy observations suggest that antisense PNA are promising candidates for a combination therapy and could be applied to improve the effectiveness of already used drugs. This could help delay or prevent the development of resistance to respective drugs.

Other essential biological processes that have been disrupted by antisense PNA, in both gram-negative and gram-positive bacteria, are DNA transcription and replication. Respectively, the *rpoD* gene encoding RNA polymerase and *gyrA* encoding DNA gyrase were targeted by PNA in several pathogens including *S. pyogenes* [111], *S. aureus, S.* Typhimurium and *Shigella flexneri* [110] (Table 2). Besides, PNA have also been used to inhibit the growth of *Mycobacterium smegmatis* [133] and the intracellular pathogen *L. monocytogenes* [112].

Interestingly, PNA targeted to specific sites of selected genes in *Bacillus subtilis* (*ftsZ* gene), *E. coli* (*murA*)*, Klebsiella pneumoniae* (*murA*), and *S.* Typhimurium (*murA, ftsZ*) in a mixed culture, selectively killed bacteria [134]. These findings open a novel opportunity for designing selective therapeutic interventions for eradication of pathogenic bacteria.

Importantly, the efficacy of the antimicrobial peptide-PNA targeting mRNA was also demonstrated in a mouse model of infection. Tan et al. showed that injection of the antisense peptide-PNA targeting the *acpP* gene significantly inhibited the growth of *E. coli* strains in mice [135]. Moreover, Abushahba et al. reported selective inhibition of *L. monocytogenes* growth in vitro, in cell culture, and in the *Caenorhabditis elegans* infection model. They also demonstrated that the PNA sequence did not adversely affect mitochondrial protein synthesis [112].

### 5.2. Ribosome as a Target for Antibacterial PNA

Many antibiotics exert their antimicrobial effects by binding to bacterial ribosome and interfering with protein synthesis (Figure 5) [136]. Three-dimensional structures of bacterial ribosomes were determined by X-ray crystallography showing that rRNA could be a promising target for the PNA oligomers. In fact, several studies demonstrated that PNA oligomers binding to the functional domains of both 23S and 16S rRNA effectively inhibited *E. coli* cell growth. For example, Good et al., used PNA oligomers to strand-invade and disrupt peptidyl transferase center (PTC) and α-sarcin loop of the 23S rRNA in the 50S ribosome subunit [137]. These PNA effectively inhibited translation in a cell-free system, as well as the growth of *E. coli* AS19 cells (Table 2). The PTC is the catalytic center of the ribosome, in which peptide bonds are formed between adjacent amino acids, so it is an essential ribosome part providing its enzymatic function. The α-sarcin loop interacts with ribosome elongation factors and is a target for cytotoxins, such as α-sarcin and ricin, which completely abolishes translation [138]. In another study, Kulik et al. inhibited *E. coli* growth with a PNA oligomer targeting a fragment of the 23S rRNA, called Helix 69 [139] (Table 2). Helix 69 forms an inter-subunit connection between the 50S and 30S ribosomal subunits and also binds some aminoglycoside antibiotics.

PNA have been also designed to bind 16S rRNA [137,140,141]. Hatamoto et al. tested PNA oligomers targeting several conserved regions of 16S rRNA using an in vitro translation assay. They found that only PNA directed against the mRNA binding site of 16S rRNA inhibited translation in a cell-free system. Furthermore, they investigated the inhibitory effect of PNA on the growth of *E. coli* K-12, *Bacillus subtilis* 168 and *Corynebacterium efficiens* YS-314 (Table 2) [140]. Importantly, besides the mRNA binding site, many other 16S rRNA regions of importance for ribosome function have been found. Górska et al., formulated the protocol that identifies regions in 16S rRNA as potential targets for sequence-specific binding and inhibition of the ribosome function [142]. The authors assessed 16S rRNA target accessibility, flexibility, and energy of strand invasion by a PNA oligomer, as well as similarity to human rRNA. They also designed and tested a PNA oligomer complementary to the 830−839 fragment of 16S rRNA of *E. coli*, which, in this particular site, is also identical in *S.* Typhimurium, and confirmed that this PNA sequence inhibited bacterial growth (Table 2) [142].

### 5.3. Other mRNA Targets

Apart from targeting the essential mRNA and rRNA, PNA oligomers were also tested against other bacterial targets, including non-essential genes. Many bacterial species form extracellular biofilms making infections extremely challenging to eradicate. Hu et al. found a PNA oligomer that effectively inhibited biofilm formation [143]. This PNA targeted the mRNA of the *motA* gene, encoding the element of the flagellar motor complex, in *Pseudomonas aeruginosa*. The biofilm formation was also hindered in *Enterococcus faecalis* by a PNA directed at the *efaA* gene, which plays an important role in the adhesion of bacteria to surfaces [144]. Besides biofilm-related genes, antibiotic resistance genes can be targeted to increase the susceptibility of resistant bacteria to antibiotics. For example, PNA aimed at a multi-drug efflux pump *cmeABC* of *Campylobacter jejuni* increased the susceptibility of this strain to ciprofloxacin and erythromycin [145].

A separate approach describes the design of a PNA-based treatment that exploits the *mazEF* and *hipBA* toxin-antitoxin systems (Figure 5) as novel targets for antisense antibacterials in a multi-drug resistant *E. coli* [146]. Many bacteria have toxin–antitoxin systems, typically composed of two genes, one encoding a toxin that targets an essential cellular process, and the other an antitoxin that counteracts the toxin activity. Równicki et al. showed that PNA can be used to modulate the expression of the toxin-antitoxin system. They found that antisense PNA effectively terminate translation of the antitoxin, causing bacterial cell death. Promisingly, the PNA oligomers did not activate cytotoxicity in mammalian cells [146].

As shown in Table 2, in the last two decades, many PNA targets in bacteria have been found and successfully verified. Still, the main PNA target in antibacterial applications of PNA is mRNA of essential genes. However, because of the complicated RNA architecture, and thus unknown mRNA fold, it is not easy to predict if the PNA-mRNA complex is formed. In addition, finding a PNA susceptible target that is present in bacteria and not present in mammalian cells is as fundamental as finding an efficient PNA carrier to bacterial cells. Contrary to other small molecule compounds, virtually any bacterial RNA can be targeted by antisense PNA, offering a limitless set to choose from. Since novel antibiotic targets are constantly searched for, they could be tested by using PNA and vice versa, PNA could also help identify them [150,151].

## 6. Conclusions

The use of sequence-specific oligonucleotides binding to natural nucleic acid targets has been a matter of extensive research, finally leading to a few FDA-approved oligonucleotide-based therapies in humans [152]. PNA as a nucleic acid mimic has been investigated for nearly 30 years. Since its first synthesis, the physicochemical properties of PNA and its interactions—especially with DNA—have been well determined. PNA oligomers have been tested in various applications, not only as antimicrobials but also as antiviral or anticancer molecules [12,153,154].

Nevertheless, the use of PNA as an antibiotic is not foreseen in the near future due to crucial limitations. The main drawback precluding the use of PNA as an antimicrobial is its lack of uptake by bacterial cells. Even though some positive examples of PNA carriers have been shown, mainly cell-penetrating peptides, we have not yet found effective PNA transporters to bacterial cells. Using modified PNA monomers could help achieve better PNA solubility and membrane permeability. Also, not only covalently bound peptides should be considered as PNA carriers, but also non-peptidic transporters. In addition, to lower the concentrations of PNA required to inhibit bacterial growth (thus PNA doses), not only an effective PNA carrier is needed but also a more PNA-susceptible target. Thus, future efforts should also focus on the search for novel PNA targets that go beyond mRNA encoding essential proteins. Despite these limitations, PNA shows promise in antibacterial studies because of its high binding affinity to RNA and strand-invasion capability. Studies highlighted in this review point to effective antibacterial PNA sequences. However, the majority of PNA sequences were tested in vitro and many questions on the PNA use in vivo still remain to be answered. Few reports have shown PNA efficacy in animal models of infection using clinically relevant doses, but most studies were performed in non-human models. Therefore, how PNA affects the interferon response and emergence of bacterial resistance remains to be seen.

It is worth noting that PNA use has already been successful in detection of bacterial pathogens. The use of PNA in diagnosis of bacterial infections has gained a lot of attention because it is critical to quickly recognize a particular pathogen to administer proper medication. The new pathogen identification platform based on the interaction of γ-PNA with double-stranded DNA shows promise in diagnostics [101]. Thus, PNA research related to bacterial applications has also focused on the diagnostic applications [155,156,157].

The number of studies related to the use of PNA in antibacterial applications is constantly growing, with PNA as a diagnostic tool for detecting pathogens paving the way. Thus, in the future the development of PNA-based antibiotics could become an alternative approach in the fight against multi-drug bacterial resistance. Other possibilities of PNA are yet to be discovered.

## Figures and Tables

**Table 1:** Structures containing PNA available in the Protein Data Bank [19] (http://www.rcsb.org).

Molecule	Structure	Method	Resolution	Includes Modified PNA Monomers	PDB ID	Ref.
PNA-PNA	duplex	X-ray	1.82 Å	bicyclic thymine analogue	1HZS	[20]
	duplex	NMR	-	-	2K4G	[21]
	duplex	X-ray	1.70 Å	-	1PUP	[22]
	duplex	X-ray	2.35 Å	-	1RRU	[23]
	duplex/triplex	X-ray	2.60 Å	-	1XJ9	[24]
	duplex	NMR	-	γ-modified PNA	2KVJ	[25]
	duplex	X-ray	1.27 Å	-	3MBS	[26]
	duplex	X-ray	2.20 Å	N-methylated PNA backbone	1QPY	[27]
	duplex	X-ray	1.05 Å	bipyridine-modified PNA	3MBU	[26]
	duplex	X-ray	1.06 Å	contains T-T mismatches	5EMG	[28]
PNA	single-stranded PNA	X-ray	1.00 Å	d-alanyl and l-homoalanyl PNA	3C1P	[29]
PNA-RNA	duplex	NMR	-	-	176D	[30]
	duplex	X-ray	1.15 Å	-	5EME	[28]
	duplex	X-ray	1.14 Å	-	5EMF	[28]
PNA-DNA	duplex	NMR	-	-	1PDT	[31]
	duplex	X-ray	1.66 Å	d-Lys based PNA	1NR8	[32]
	duplex	X-ray	1.60 Å	γ-modified PNA	3PA0	[33]
PNA-DNA-PNA	triplex	X-ray	2.50 Å	HIS-GLY-SER-SER-GLY-HIS-linker	1PNN	[34]

**Table 2:** Minimal inhibitory concentrations (MICs) determined for (KFF)_3_K-PNA conjugates targeted at various genes. The MIC values provided in the table are the lowest determined MICs in each case.

Target	Function	Bacteria	MIC * (μM)	Reference
**mRNA of essential genes**				
*acpP*	fatty acid biosynthesis	*Brucella suis* 1330	30 **	[128]
		*Escherichia coli* K-12	0.6	[11]
		*Haemophilus influenza*	0.6	[129]
		*Pseudomonas aeruginosa* PAO1	2	[130]
*hmrB*		*Staphylococcus aureus* RN4220	10	[122]
*fabI*		*Escherichia coli* K-12	3	[131]
		*Staphylococcus aureus* RN4220	15	
*folA*	folate biosynthesis	*Escherichia coli* AS19	2.5	
*folP*		*Escherichia coli* AS19	2.5	
*gyrA*	DNA replication	*Acinetobacter baumanii* CY-623	5	[147]
		*Brucella suis* 1330	30	[128]
		*Klebsiella pneumoniae*	20	[148]
		*Staphylococcus aureus* RN4220	10	[131]
		*Streptococcus pyogenes*		[111]
*rpoD*	DNA transcription	*Escherichia coli* (ESBL+)	6.2	[110]
		*Klebsiella pneumoniae* (ESBL+)	30	[110]
		*Listeria monocytogenes* ATCC 19114	2 ***	[112]
		*Salmonella enterica* serovar Typhimurium LT2	15 ***	[149]
		*Shigella flexneri* (MDR)	5	[110]
		*Staphylococcus aureus* ATCC29213	6.2	[108]
*murA*	cell-wall biogenesis	*Escherichia coli* DH10B	2.4	[134]
		*Klebsiella pneumoniae* ATCC 700721	2.5	
		*Salmonella enterica* serovar Typhimurium LT2	1.2	
*ftsZ*	cell division	*Bacillus subtilis* 168	4	[134]
		*Salmonella enterica* serovar Typhimurium LT2	2.5	
*inhA*	mycolic acid biosynthesis	*Mycobacterium smegmatis* 155	<5	[133]
**rRNA**				
PTC	peptidyl transferase center 23S rRNA	*Escherichia coli* K-12	50 ***	[137]
*a-sarcin loop*	binds elongation factor G (EF-G) 23S rRNA	*Escherichia coli* K-12	50 ***	
Helix 69	forms connection between ribosomal subunits	*Escherichia coli* K-12	15	[139]
mRBS	mRNA binding site 16S rRNA	*Corynebacterium efficiens*	2	[140]
		*Bacillus subtilis*	5	
		*Escherichia coli* K-12	10	
830−839 16S RNA	part of IF3 binding site 16S rRNA	*Escherichia coli* K-12	15	[142]
830−839 16S RNA	part of IF3 binding site 16S rRNA	*Salmonella enterica* serovar Typhimurium LT2	5	
**Other mRNA targets**				
*motA*	biofilm formation	*Pseudomonas aeruginosa* PAO1	1	[143]
*cmeABC*	multidrug efflux transporter	*Campylobacter jejuni*	-	[145]
*mazE*	antitoxin MazE	*Escherichia coli* WR3551/98	16	[146]
*hipB*	antitoxin HipB	*Escherichia coli* WR3551/98	16	
*thyA*	thymidylate synthase	*Escherichia coli* WR3551/98	16	
*gltX*	glutamyl-tRNA synthetase	*Escherichia coli* WR3551/98	2	

* MICs were tested in Mueller–Hinton broth, unless otherwise stated ** MICs were tested in Tryptic Soy broth *** Inhibition assays performed using solid LB/agar plates.
